# Sequential mutations associated with adaptation of human cytomegalovirus to growth in cell culture

**DOI:** 10.1099/vir.0.018994-0

**Published:** 2010-06

**Authors:** Derrick J. Dargan, Elaine Douglas, Charles Cunningham, Fiona Jamieson, Richard J. Stanton, Katarina Baluchova, Brian P. McSharry, Peter Tomasec, Vincent C. Emery, Elena Percivalle, Antonella Sarasini, Giuseppe Gerna, Gavin W. G. Wilkinson, Andrew J. Davison

**Affiliations:** 1 MRC Virology Unit, Institute of Virology, University of Glasgow, Church Street, Glasgow G11 5JR, UK; 2 Department of Medical Microbiology, Tenovus Building, School of Medicine, Cardiff University, Heath Park, Cardiff CF14 4XX, UK; 3 Centre for Virology, Division of Infection and Immunity, Royal Free and University College Medical School, Rowland Hill Street, Hampstead, London NW3 2QG, UK; 4 Servizio di Virologia, Fondazione IRCCS Policlinico San Matteo, 27100 Pavia, Italy

## Abstract

Mutations that occurred during adaptation of human cytomegalovirus to cell culture were monitored by isolating four strains from clinical samples, passaging them in various cell types and sequencing ten complete virus genomes from the final passages. Mutational dynamics were assessed by targeted sequencing of intermediate passages and the original clinical samples. Gene RL13 and the UL128 locus (UL128L, consisting of genes UL128, UL130 and UL131A) mutated in all strains. Mutations in RL13 occurred in fibroblast, epithelial and endothelial cells, whereas those in UL128L were limited to fibroblasts and detected later than those in RL13. In addition, a region containing genes UL145, UL144, UL142, UL141 and UL140 mutated in three strains. All strains exhibited numerous mutations in other regions of the genome, with a preponderance in parts of the inverted repeats. An investigation was carried out on the kinetic growth yields of viruses derived from selected passages that were predominantly non-mutated in RL13 and UL128L (RL13^+^UL128L^+^), or that were largely mutated in RL13 (RL13^−^UL128L^+^) or both RL13 and UL128L (RL13^−^UL128L^−^). RL13^−^UL128L^−^ viruses produced greater yields of infectious progeny than RL13^−^UL128L^+^ viruses, and RL13^−^UL128L^+^ viruses produced greater yields than RL13^+^UL128L^+^ viruses. These results suggest strongly that RL13 and UL128L exert at least partially independent suppressive effects on growth in fibroblasts. As all isolates proved genetically unstable in all cell types tested, caution is advised in choosing and monitoring strains for experimental studies of vulnerable functions, particularly those involved in cell tropism, immune evasion or growth temperance.

## INTRODUCTION

Human cytomegalovirus (HCMV; species *Human herpesvirus 5*) can infect most major organs and a wide range of cell types (Bissinger *et al.*, 2002; Plachter *et al.*, 1996; Sinzger *et al.*, 1995, 2008a). This broad tropism is lost during adaptation of the virus to fibroblasts in cell culture (Hahn *et al.*, 2004; Kahl *et al.*, 2000; MacCormac & Grundy, 1999; Revello *et al.*, 2001; Sinzger *et al.*, 1999; Waldman *et al.*, 1989, 1991). Adaptation to fibroblasts is associated strongly with mutations in a region encompassing three small genes at nt 176309–178146 in reference strain Merlin (GenBank accession no. AY446894). These genes are UL128, UL130 and UL131A, which together are termed the UL128 locus (UL128L). The mutations are predicted to ablate gene functions, and include nucleotide substitutions that introduce in-frame translational termination codons or compromise splicing, small insertions or deletions (indels) that introduce frameshifts into coding regions, and larger deletions or inversions that affect one or more genes.

UL128L was originally described as containing four unspliced coding regions: UL128, UL129, UL130 and UL131 (Chee *et al.*, 1990). Redefinition based on comparative genomics led instead to three genes: UL128 (three exons), UL130 (unspliced) and UL131A (two exons) (Davison *et al.*, 2003a). This arrangement was confirmed by transcript mapping (Akter *et al.*, 2003; Hahn *et al.*, 2004). Each gene was predicted to encode a small protein that transits through the secretory pathway (Akter *et al.*, 2003; Hahn *et al.*, 2004). The UL128 protein has sequence similarity to CC chemokines (Akter *et al.*, 2003; Hahn *et al.*, 2004), and the UL130 protein contains a putative CX chemokine fold (Novotny *et al.*, 2001; Wyrwicz & Rychlewski, 2007). The observation that virus passaged in fibroblasts is usually mutated in only one of the UL128L genes implies that the proteins act in concert (Akter *et al.*, 2003; Hahn *et al.*, 2004), and the implication that this function is detrimental to growth of HCMV in these cells has found experimental support (Adler *et al.*, 2006; Wang & Shenk, 2005a). In contrast, all three genes are known to be essential for growth of HCMV in endothelial cells (Hahn *et al.*, 2004) and dendritic cells (Gerna *et al.*, 2005), and at least one (UL131A) for growth in epithelial cells (Wang & Shenk, 2005a). These observations are consistent with an involvement of UL128L in cell tropism, specifically for infection of non-fibroblast cells (Sinzger, 2008; Sinzger *et al.*, 2000). The UL128L proteins are present in virions (Adler *et al.*, 2006; Patrone *et al.*, 2005; Wang & Shenk, 2005b), where they form a complex with virus glycoproteins H and L (gH–gL), as an alternative to glycoprotein O (gO) in the gH–gL–gO complex, for entry into non-fibroblast cells via endocytosis and low pH-dependent fusion (Ryckman *et al.*, 2006, 2008b) or direct fusion with the plasma membrane (Patrone *et al.*, 2007). This key role for the gH–gL–UL128L complex is supported by the observation that antibodies against any of the UL128L proteins inhibited infection of endothelial and epithelial cells, but not fibroblasts (Adler *et al.*, 2006; Macagno *et al.*, 2010; Wang & Shenk, 2005b). Also, endogenous expression of the gH–gL–UL128L complex rendered epithelial cells, but not fibroblasts, refractory to infection by viruses in which UL128L was intact, thus implying that specific receptors may exist on non-fibroblast cells (Ryckman *et al.*, 2008a).

Mutations in other regions of the genome have been reported for strains passaged in fibroblasts, and most appear to be sporadic. However, several mutations affect members of the RL11 gene family, which has 14 related members (RL5A, RL6, RL11, RL12, RL13, UL1, UL4, UL5, UL6, UL7, UL8, UL9, UL10 and UL11), most of which are predicted to encode class I membrane glycoproteins (Chee *et al.*, 1990; Davison *et al.*, 2003b). Lesions take the form of termination, frameshift or deletion mutations, and have been identified in RL13 (nt 11189–12070 in reference strain Merlin), RL5A, RL6 and UL9 (Cunningham *et al.*, 2010; Dolan *et al.*, 2004).

The observations described above depend largely on targeted regions in the genomes of various HCMV strains. We set out to paint a full picture of what happens dynamically to entire HCMV genomes when adapted from clinical samples to cell culture. Our aim was to identify patterns of mutation that occur during adaptation and to assess whether HCMV is genetically more stable in non-fibroblast than in fibroblast cells.

## RESULTS

### Genomes sequenced completely

As multiple HCMV strains are common in clinical samples, 14 isolates were tested during isolation by PCR amplification of hypervariable genes UL146 and UL139, followed by sequencing the inserts in several plasmids (Bradley *et al.*, 2008). The epidemiologically distinct AF1, U8 and U11 isolates (see Methods) were each found to consist of a single strain and were chosen for further passaging. The analysis was complemented by data from a fourth isolate (VR1814; see Methods) that had been passaged earlier. All sequence data obtained were fully in accordance with the presence of a single, unique strain in each isolate.

**Fig. 1.**
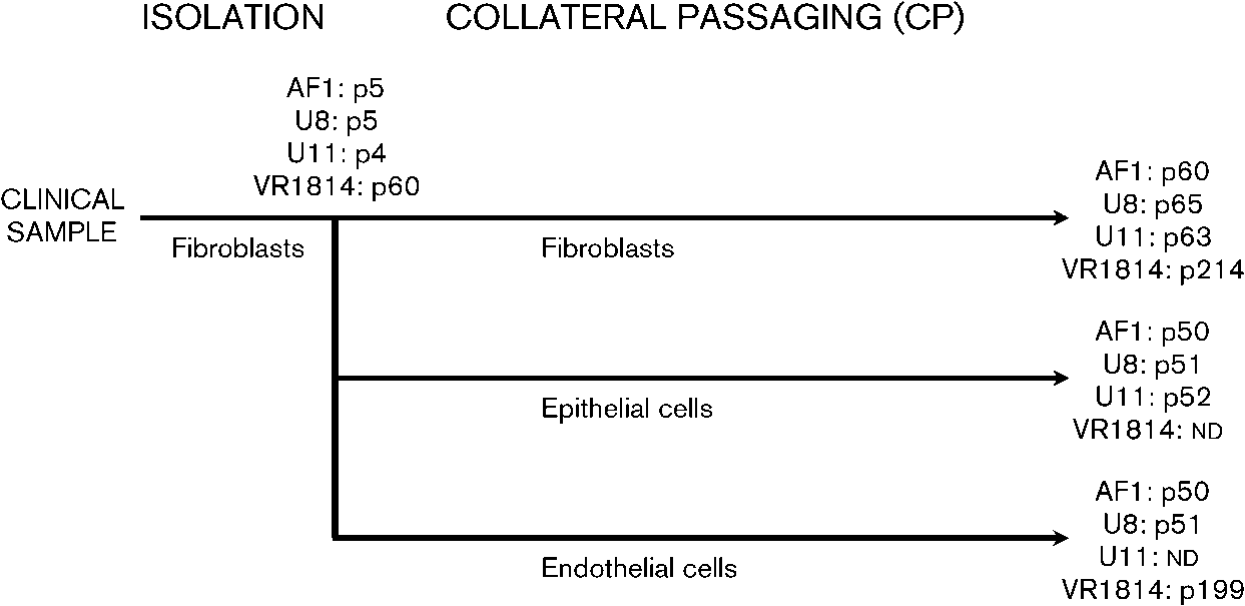
Scheme for isolation of HCMV strains from clinical samples in fibroblasts and CP in fibroblast, epithelial and endothelial cells. The passage number (p) at which CP commenced and terminated is shown for each strain. The final passage numbers for fibroblasts include those involved in virus isolation, whereas those for non-fibroblast cells do not. nd, Not done.

The strategy for adapting strains AF1, U8 and U11 to growth in cell culture is outlined in Fig. 1. The strains were isolated from clinical samples by using human fetal foreskin fibroblasts (HFFF-2) grown in shell vials, followed by expansion of the infected cells by a limited number (four/five) of passages. Infectivity was transferred in a single passage by co-culture of infected HFFF-2 cells with hTERT-immortalized human retinal pigmented epithelial (RPE-1) cells or primary human umbilical vein endothelial cells (HUVECs). Cell-free virus, obtained by sonication of the co-cultures, was then used to establish passage series in epithelial and endothelial cells, and passaging in fibroblasts was also continued. The strains were subjected weekly to collateral passaging (CP) in the three cell types by co-culture until at least passage 50 (p50), except that passaging of strain U11 in endothelial cells failed at an early stage for unknown reasons. Strain VR1814 was passaged by using similar methods, but much more extensively: 60 times in fibroblasts (isolation) followed by CP, attaining p214 in fibroblasts and p199 in endothelial cells (Fig. 1). In the fibroblast series, passage numbering started with the first isolation passage after growth in shell vials. In the epithelial and endothelial cell series, numbering started with the initiation of CP. Passage descriptions were abbreviated according to strain name, cell type (F, fibroblasts; R, epithelial cells; E, endothelial cells) and passage number. Thus, passage 35 of strain U8 in epithelial cells was denoted U8Rp35.

At the termination of CP, the complete HCMV genomes in the final passages were sequenced, yielding a total of ten sequences: AF1Fp60, AF1Rp50 and AF1Ep50; U8Fp65, U8Rp51 and U8Ep51; U11Fp63 and U11Rp52; and VR1814Fp214 and VR1814Ep199.

### Genomes sequenced partially

Two further passage series (II and III) of strain U11 were carried out in fibroblasts, in addition to the original series (I). Series II involved initiating CP with the virus isolated previously during series I (p4), whereas series III commenced with reisolation of virus from the clinical sample. RL13 and UL128L were sequenced from the final passages of series II and III (p51 and p40, respectively).

Strain VR1814 is the parent of the bacterial artificial chromosome (BAC) FIX-BAC, which was generated by cloning a genome present at p46 in fibroblasts (Hahn *et al.*, 2002) and sequenced (Murphy *et al.*, 2003). A virus (RVFIX7) reconstituted from FIX-BAC that had been passaged approximately five times in fibroblasts (Hahn *et al.*, 2002) was treated with anti-mycoplasma agents during five further passages in fibroblasts. This virus was then subjected to CP by 117 passages in fibroblasts or 94 passages in endothelial cells to derive RVFIXFp117 and RVFIXEp94. Sequence data were derived for the region containing RL1–UL20 (nt 1772–28092, which includes RL13) for VR1814Fp22, VR1814Fp48, FIX-BAC, RVFIXFp117 and RVFIXEp94, and for the region containing UL122–UL150 (nt 168563–194150, which includes UL128L) for VR1814Fp48, FIX-BAC, RVFIXFp117 and RVFIXEp94.

### Identification of mutations

Mutations in a particular strain were identified from differences between the genome sequences at the final passages, whether or not they were predicted to affect gene functions. The mutated and wild-type versions were differentiated from each other in two ways: directly on the basis of targeted data from earlier passages and the clinical samples, and indirectly by reference to sequence alignments for other HCMV strains. Most mutations consisted of substitutions or indels, but several sizeable deletions were also detected. Mutations that did not result in differences between the final sequences, but that had been present in the clinical sample or had arisen during isolation, would not have been detected except where they potentially ablated gene functions. Also, extensive deletions or rearrangements present in a proportion of, but not all, genomes might not have been detected. Inspection of sequence traces facilitated the identification not only of predominant mutations, but also of mutations represented in a significant minority (>10 %) of the HCMV genome population. The dynamics by which mutations arose were monitored by reference to targeted sequence or PCR data from earlier passages or the clinical samples. The approaches used did not permit the complete absence of a particular sequence (mutant or wild-type) to be inferred.

The mutations detected in the four strains are summarized in Tables 1–4. Mutations were detected at each stage: a few in the clinical samples (not included in Tables 1–4, but described below), some during isolation (e.g. the substitution in UL72 at nt 106148 of strain AF1; Table 1), and the majority during CP (e.g. the substitution in UL72 at nt 105981 of strain AF1; Table 1). It is notable that events seem to have moved at a slower pace in epithelial cells than in fibroblasts or endothelial cells.

The HCMV genome has the structure *ab*-U_L_-*b*′*a*′*c*′-U_S_-*ca*, where U_L_ and U_S_ denote long and short unique regions and *ba*/*b*′*a*′ and *ca*/*c*′*a*′ indicate inverted repeats flanking the unique regions. The region most prone to mutations was the *b*/*b*′ inverted repeat, with a lower frequency in the flanking regions of the *a*/*a*′ and *c*/*c*′ inverted repeats. Owing to the locations of the PCR products utilized for sequencing, mutations in separate copies of the inverted repeats could be evaluated for the whole of *b*/*b*′ and *a*/*a*′ and the proximal part of *c*/*c*′, and were found to be approximately equimolar. This observation is consistent with recombination between the repeats during virus DNA replication.

### Strain AF1

RL13 mutants were detected in fibroblasts (nt 11460 and 11667; the same genome population), epithelial cells (nt 11488–11489) and endothelial cells (nt 10984) (Table 1). Mutation of UL128L (UL130; nt 177046) occurred only in fibroblasts, and was first detected in a later passage (p20) than that in RL13 (p15). Seven clustered substitutions occurred in UL123 (nt 172215–173747; the same genome population) in endothelial cells. The strain AF1 genome in the clinical sample was mutated in two genes: RL6 containing an in-frame translational termination codon due to a C to A substitution at nt 5936, and UL9 containing a frameshift due to an additional A residue at nt 17079.

### Strain U8

RL13 mutants were detected in fibroblasts (nt 11924), epithelial cells (nt 11269–11270) and endothelial cells (nt 11890) (Table 2). Mutation of UL128L (UL131A; nt 178000) occurred only in fibroblasts, and was first detected in a later passage (p18) than that in RL13 (p16). Four clustered substitutions occurred in fibroblasts in the region containing UL142 and UL141 (nt 182822–184383; the same genome population). Complex, unresolved sequence heterogeneity (not included in Table 2) was evident as various deletions in the region containing the *b*–U_L_ junction in fibroblasts and possibly also epithelial and endothelial cells. No obvious mutations were identified in the strain U8 clinical sample.

### Strain U11

Strain U11 was passaged successfully only in fibroblast and epithelial cells (Table 3, series I). An RL13 mutation (nt 11378) was detected in both cell types. Mutation of UL128L (UL128; nt 176252 and 176307–176311; different genome populations) was detected in fibroblasts, and was first detected in a later passage (p15) than that in RL13 (p8). The same mutations were also detected at low levels in epithelial cells. Generation of the five concurrent substitutions at nt 176307–176311 probably occurred in a single event involving switches of replication polarity or recombination in a hairpin (ACGCCGTCAAGAACGGCGT; inverted repeat underlined). The strain U11 genome in the clinical sample was mutated in a single gene (UL9) due to a frameshifting 23 bp deletion near the 3′ end (between nt 17102 and 17103). Several examples of mutations present in both final passages were detected, including those in RL13 and UL128, implying that they may have arisen during isolation. The reason for the apparently faster rate of mutant selection in strain U11 than in strains AF1 and U8 is not known.

To investigate whether the mutations detected in both final passages had been present in the clinical sample or had originated during isolation, two additional passage series were carried out in fibroblasts. In series II, in which CP was initiated with the virus isolated during series I, three mutations in RL13 were detected (different genome populations) and one in UL128L (again, UL128). One of the RL13 mutations (nt 11378) reiterated a mutation detected in series I. In series III, which commenced with reisolation of virus from the clinical sample, two mutations in RL13 were detected (different genome populations) and one in UL128L (again, UL128), none corresponding to mutations observed in series I and II. In a further investigation, PCR products from the RL13 and UL128 regions were generated from the clinical sample. None of the 48 plasmids generated from each product contained any of the mutations detected in series I, II and III.

### Strain VR1814

Compared with strains AF1, U8 and U11, strain VR1814 had been subjected to more extensive passaging during isolation and CP (Fig. 1) and more mutations were observed (Table 4). However, the analysis was limited in that the clinical sample was not available, and it was possible to differentiate the mutated and wild-type versions of a particular sequence only indirectly, by reference to sequence alignments for other HCMV strains.

RL13 mutants were present in both fibroblasts and endothelial cells (nt 10999–12018 and 11775; different genome populations), and these were first detected during isolation (p48; Table 4). A frameshift-inducing mutation in UL128L (UL131A; nt 177905) in fibroblasts was first detected during CP (p95). In addition, non-synonymous substitutions in UL128L (UL130) that left the coding region intact were detected in fibroblasts (p48; nt 177250) and endothelial cells (p199; nt 176856). Complex, unresolved sequence heterogeneity (not included in Table 4) was detected in the *b*/*b*′ and *a*/*a*′ inverted repeats. No obvious mutations in the clinical sample were inferred. FIX-BAC and derived viruses RVFIXFp117 and RVFIXEp94 contained one of the RL13 mutations (nt 11775) and one of the UL128L (UL130) mutations (nt 177250).

Passages of strain VR1814 in fibroblasts were assessed for endotheliotropism (p55, p154 and p214) and leukocyte transfer (p22, p48, p55, p95, p121, p143, p154, p179, p194 and p214). Endotheliotropism was lost between p55 and p154, and remained negative at p214. Leukocyte transfer was lost between p95 (2000 positive cells per 2×10^5^ leukocytes) and p154 (no positive cells), with reductions registered at p121 (540 positive cells) and p143 (65 positive cells) and leukocyte transfer remaining negative at p214. These findings are consistent with detection of the UL128L (UL131A) mutation at p95 and its predominance at p121 (Table 4). Both RVFIXFp117 and RVFIXEp94 tested positive for leukocyte transfer and endotheliotropism.

### Growth properties

The passage series derived for strains AF1, U8 and U11 provided a means for obtaining virus stocks that were largely non-mutated in RL13 and UL128L (RL13^+^UL128L^+^), mutated in RL13 (RL13^−^UL128L^+^) or mutated in both locations (RL13^−^UL128L^−^). The process of generating stocks was protracted, taking approximately 2 weeks for RL13^−^UL128L^−^ viruses, 8 weeks for RL13^−^UL128L^+^ viruses and 10 weeks for RL13^+^UL128L^+^ viruses. To distinguish between the ability of cell-free virus in these stocks to initiate infection and to form a plaque, titrations in fibroblasts were either subjected to immunofluorescence (IF) analysis or stained for plaque counting. Regardless of strain, the ratio of the number of IF-positive cells to that of plaques was of the order of 100 for RL13^+^UL128L^+^ viruses, 10 for RL13^−^UL128L^+^ viruses, and <10 for RL13^−^UL128L^−^ viruses.

**Fig. 2.**
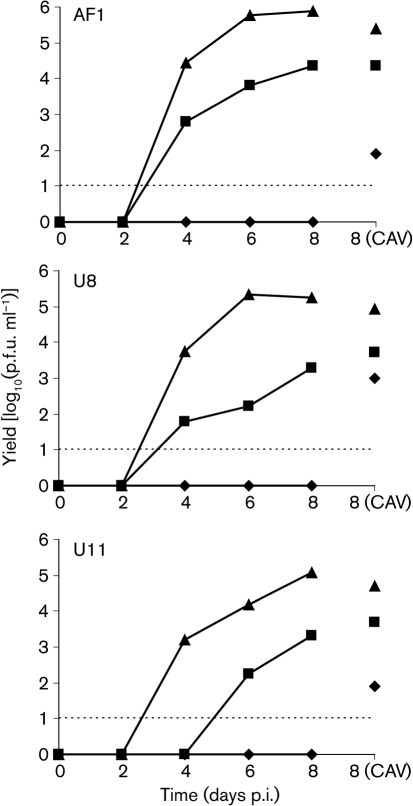
Kinetics of virus growth in fibroblasts of RL13^+^UL128L^+^ (⧫), RL13^−^UL128L^+^ (▪) and RL13^−^UL128L^−^ (▴) viruses derived from strains AF1, U8 and U11. The connected points show CRV titres and the unconnected points on the right show CAV titres. Values below the limit of detection (dotted line) are assigned to the *x*-axis.

Kinetic-yield experiments in fibroblasts (Fig. 2) showed that the RL13^−^UL128L^−^ viruses produced >10-fold more cell-released virus (CRV) than RL13^−^UL128L^+^ viruses, which in turn produced >100-fold more CRV than RL13^+^UL128L^+^ viruses. Indeed, no CRV was detected from RL13^+^UL128L^+^ viruses. All viruses produced measurable amounts of cell-associated virus (CAV) at 8 days post-infection (p.i.), and the relative order was the same as that for CRV.

## DISCUSSION

### Isolation and passaging of strains

Strains AF1, U8 and U11 were isolated by a few passages in fibroblasts, thus subjecting them to fibroblast-associated selection pressures prior to CP. Indeed, mutations that apparently arose at this stage were identified for each strain, in particular strain U11. Isolation in fibroblasts was necessary because repeated attempts to isolate strains AF1, U8 and U11 directly in epithelial and endothelial cells were unsuccessful. This failure is consistent with the far greater ease with which HCMV may be isolated in fibroblasts than in other cell types (Gerna *et al.*, 2002b). This phenomenon may be connected in part with the neutralizing activity of antibodies against the UL128L proteins (Gerna *et al.*, 2008), which are potentially bound to virus particles in the inoculum. Historically, strains in clinical samples have been isolated by passage in fibroblasts and then transferred to other cell types either by co-culture of infected fibroblasts and target cells or by infection of target cells with cell-free virus produced by disruption of fibroblast cultures (Gerna *et al.*, 2000, 2002b; MacCormac & Grundy, 1999; Sinzger *et al.*, 1999; Waldman *et al.*, 1991). We utilized co-culture for CP, which would have applied less selection in favour of virus release than passage of cell-free virus.

### Mutations in RL13 and UL128L

In strains AF1 and U8, RL13 mutated in fibroblast, epithelial and endothelial cells (at a different location in each cell type), and UL128L (UL130 and UL131A, respectively) mutated only in fibroblasts. In strain U11, for which endothelial-cell passaging failed, mutations in RL13 and UL128L (UL128) were detected in both fibroblasts and epithelial cells, indicating that they had occurred during isolation. Consistent with the known requirement for UL128L in non-fibroblast cells, the UL128L mutations in strain U11 were predominant in fibroblasts, but present at low levels in epithelial cells, where they were presumably maintained by complementation. In strain VR1814, RL13 mutated during isolation, and the UL128L (UL131A) mutant was detected later in fibroblasts, but not endothelial cells. In all four strains, mutation of RL13 in fibroblasts was detected before that of UL128L, and mutations in both loci quickly became predominant.

The loss of UL128L and RL13 fits with previous observations on the presence of mutations in these loci in strains passaged in fibroblasts. However, in contrast to UL128L, RL13 mutated not only in fibroblasts, but also in epithelial and endothelial cells. Moreover, kinetic-yield experiments suggested that RL13 and UL128L suppress growth in fibroblasts at least partially independently, pointing to the involvement of different functional pathways. CP-derived viruses were used to investigate growth properties, but were of limited utility because they contained small amounts of alternative genotypes, and other mutations were also present in various proportions. Nonetheless, the similar growth properties obtained for viruses derived from strains AF1, U8 and U11 support the view that whether RL13 and UL128L were mutant or non-mutant was the main factor affecting differences in growth properties. In accord, a collaborative study has shown that virus derived from a strain Merlin BAC containing the wild-type gene complement (RL13^+^UL128L^+^) invariably mutated in RL13 in both fibroblasts and epithelial cells, with UL128L mutants being detected later only in fibroblasts (R. J. Stanton, unpublished data). Future investigations into the growth properties of RL13^+^UL128L^+^ virus are likely to be made to best effect using the Merlin system.

The number of passages required for RL13 and UL128L (UL131A) to mutate in strain VR1814 appeared to be greater than that for the other strains. The observations that mutation of UL128L (UL131A) was detected at a particularly late stage (p95) and that the FIX-BAC-derived viruses (RVFIXFp117 and RVFIXEp94; both ostensibly RL13^−^UL128L^+^) did not mutate during passage in fibroblasts suggest genetic compensations elsewhere. In this connection, it is notable that strain VR1814 grown in fibroblasts had an additional, earlier mutation in UL128L, specifically a non-synonymous substitution in UL130. This mutation was inherited by FIX-BAC and the viruses derived from it. It is possible that this mutation reduced the selection pressure for further mutations in UL128L during growth in fibroblasts, whilst permitting the retention of a degree of endotheliotropism and ability to transfer to leukocytes. This might explain why UL128L mutations engineered into FIX-BAC-derived viruses did not result in significantly enhanced growth in fibroblasts in kinetic-yield experiments (Hahn *et al.*, 2004; commented on by Wang & Shenk, 2005a). In a similar vein, strain TB40/E (Sinzger *et al.*, 2008b) has a unique non-synonymous substitution in UL130 that might account for its ability to grow in both fibroblasts and endothelial cells (Dolan *et al.*, 2004). These comments prompt caution about assuming wild-type status for UL128L in fibroblast-passaged HCMV strains (as viruses or BACs) that retain the ability to grow in non-fibroblast cells.

It is not fully understood why UL128L is detrimental to growth in fibroblasts, although inhibition of virion release has been proposed (Adler *et al.*, 2006; Wang & Shenk, 2005a). Preliminary studies indicate that the RL13 product is a 100 kDa type I transmembrane glycoprotein (K. Baluchova, unpublished data), but its function and the reason for its inhibitory properties are unknown. It is possible that its suppressive effects in cell culture reflect similar properties operating *in vivo*, where HCMV persistence might be facilitated by mechanisms that temper virus replication. Alternatively, the effect might result from an interference with replication in cell culture that does not occur *in vivo*. Nonetheless, there remains a striking contrast between the enfeebled growth properties of RL13^+^UL128L^+^ virus in cell culture and the ability of HCMV to replicate well in clinical situations (Emery *et al.*, 1999).

### Mutations in UL145–UL140

After passaging in endothelial cells, strain U8 had a 551 bp deletion in UL144 that also had the potential for affecting UL145 transcription (Table 2). After passage in fibroblasts and epithelial cells, strain U11 had a single nucleotide insertion that caused a frameshift in UL141 (Table 3). After passage in fibroblasts and endothelial cells, strain VR1814 had a 3173 bp deletion that affected UL142, UL141 and UL140 and possibly also UL144 transcription (Table 4). Only strain AF1 was unaffected in this region. Lesions usually took the form of deletions, suggesting that more than one gene was selected against, although not as strongly as RL13 and UL128L.

Mutations in a region containing five genes (UL145, UL144, UL142, UL141 and UL140) have been identified for strain AD169 varUC in fibroblasts (ablating UL144, UL142, UL141 and perhaps affecting UL140 transcription), strain TB40/E in endothelial cells (UL141, with a derivative additionally compromised in UL145 and UL144) and strain VR3216B in endothelial cells (UL142) (Bradley *et al.*, 2009; Sinzger *et al.*, 2008b; Tomasec *et al.*, 2005; GenBank accession no. GQ222018). In addition, the genomes of the most widely used HCMV strains, AD169 (varUK and varATCC) and Towne (varS), each lack a sizeable region (U_L_/*b*′; 15 or 19 genes, respectively) that includes UL145–UL140 (Bradley *et al.*, 2009; Cha *et al.*, 1996; Davison *et al.*, 2003a; Murphy *et al.*, 2003). It is possible that these extensive deletions represent one end of a spectrum of mutations whose key effect is to disable UL145–UL140.

### Other mutations

All four strains exhibited numerous mutations in the inverted-repeat regions. The dynamics by which these mutations arose was not determined. Their frequent occurrence might be promoted in part by recombination, but other factors are probably important, as the major part of *c*/*c*′ was not affected. Many of the mutations in other regions of the genome were non-synonymous substitutions, which suggests that at least some were selected because they provided growth advantages. A cluster of mutations in the same genome population was evident for strains AF1 and U8, in UL123 for the former and in the region of UL142 and UL141 for the latter. These clusters may have resulted from local, error-prone replication of a genome whose progeny was then selected.

### Origins of mutations

The passaging experiments show that the HCMV genome was unstable during adaptation to cell culture, regardless of virus strain and cell type. However, the identification of mutations that apparently arose during isolation raised the question of whether they might have been present at low levels in the clinical sample. This possibility is technically difficult to rule out. If it is the case, the data from reisolation of strain U11 indicate the existence of a swarm of different mutants, each present at a very low level in the clinical sample. However, there was no evidence from sequence traces for the prior existence of any mutation. Moreover, none of the RL13 or UL128 mutants in strain U11 was detected in sets of PCR-derived plasmid clones generated from the clinical sample, thus excluding an approximate frequency per mutation of >2 %.

In contrast to the lack of evidence for the presence of the mutations described above in the clinical samples, a small number of mutations (in RL6 and UL9) were identified as characterizing the entire populations of HCMV genomes in the clinical samples from which strains AF1 and U11 were derived. Their identification strengthens the view that HCMV mutants lacking functions, particularly in the RL11 gene family, exist in clinical settings (Cunningham *et al.*, 2010; Sekulin *et al.*, 2007).

## METHODS

### Clinical samples.

Clinical samples were obtained in accordance with the relevant national ethical guidelines. Strains AF1, U8 and VR1814 originated from Pavia, Italy, and strain U11 from London, UK. Strain AF1 was derived from amniotic fluid from a pregnant woman infected with HCMV, strains U8 and U11 from urine from infants infected congenitally with HCMV, and strain VR1814 from the cervical secretions of a pregnant woman with a primary HCMV infection.

### Cell lines.

Strains AF1, U8 and U11 were isolated and passaged in Glasgow, UK, in three different cell lines (Fig. 1). HFFF-2 cells (ECACC 86031405) were grown in Dulbecco's modified Eagle's medium (DMEM)/10 % (v/v) fetal calf serum. hTERT-immortalized RPE-1 cells (Clontech C4000-1) were grown in DMEM/Ham's F-12 supplement/10 % (v/v) fetal calf serum. Primary HUVECs were prepared from umbilical cords from consenting anonymous volunteers attending the Queen Mother's Maternity Hospital, Glasgow, UK. Cords were obtained in accordance with National Health Service ethical guidelines. HUVECs were grown using the EGM-2 BulletKit system (Lonza) without heparin and subjected to IF using mouse antibody MCA 127T (Serotec) to confirm the presence of the von Willebrand factor. Each cell line was checked regularly for mycoplasma contamination by using a Minerva Biolabs VenorGeM mycoplasma detection kit (Cambio). Strain VR1814 was isolated and passaged similarly in Pavia, Italy, using human embryo lung fibroblasts (VO cells) and primary HUVECs.

### Isolation of HCMV strains.

The AF1, U8 and U11 clinical samples were diluted 1 : 3 with DMEM and 500 μl was inoculated onto fibroblasts grown on glass coverslips in shell vials. Following centrifugation at 450 ***g*** for 30 min at 18 °C, the cultures were incubated overnight at 37 °C. The cultures were then trypsinized and the infected cells were expanded by passaging, reaching the stage of 75 cm^2^ cell-culture flasks. Plaques first appeared for strains AF1, U8 and U11 at p2, p4 and p2, respectively, and infectivity was transferred to epithelial and endothelial cells by co-culture with infected fibroblasts at p5, p5 and p4, respectively. Co-cultures were harvested by scraping the cells into the medium and disrupting them by ultrasonic treatment. Cell-free virus was then used to infect fresh monolayers of epithelial and endothelial cells, thus avoiding overgrowth by fibroblasts. Passaging in fibroblasts was also continued. Strain VR1814 was isolated and transferred similarly, except that it was passaged 60 times in fibroblasts (isolation) and then transferred to endothelial cells or continued in fibroblasts for CP.

### Passaging of HCMV strains.

Strains AF1, U8 and U11 were subjected to CP (>50 times) in fibroblast, epithelial and endothelial cells on a weekly basis by co-culture of infected cells with uninfected cells. Split ratios (infected : uninfected cells) depended on the extent of cytopathic effect (CPE). Typically, they were 1 : 2 to 1 : 4 for epithelial and endothelial cells and 1 : 2 to 1 : 250 for fibroblasts. At most passages, aliquots of the infected cultures were withdrawn for storage in liquid nitrogen, initiating the next passage, or extracting infected-cell DNA by using a FlexiGene kit (Qiagen). Following isolation, strain VR1814 was subjected to CP a further 154 times (i.e. a total of 214 times) in fibroblasts and 199 times in endothelial cells, and material from certain passages was retained.

### DNA sequencing.

The HCMV genome present in infected-cell DNA at each final passage was sequenced in its entirety by a PCR-based approach involving the use of a large library of conserved, HCMV-specific primers for generating and sequencing a set of overlapping PCR products (Cunningham *et al.*, 2010). Data were obtained in the form of sequence traces from an Applied Biosystems 3730 instrument at the BHF Glasgow Cardiovascular Research Centre (University of Glasgow, UK). The processed data were assembled by using the Staden Pregap4 and Gap4 programs, and the traces were inspected visually throughout. Most PCR products were sequenced directly, but some were cloned into four to six plasmids. In some instances, clones were utilized to determine whether closely located mutations were present in the same genome population. Targeted sequencing or PCR (for deletions) of HCMV DNA in selected lower passages or the clinical specimen was also carried out.

### Production of HCMV stocks.

Infected cells at passages in which RL13^+^UL128L^+^, RL13^−^UL128L^+^ or RL13^−^UL128L^−^ viruses were predominant were recovered from liquid nitrogen and seeded on monolayers of the same cell line. Infectivity was amplified by several rounds of co-culture with uninfected cells and, in the case of RL13^+^UL128L^+^ and RL13^−^UL128L^+^ viruses, by a final 1 : 1 co-culture step with fibroblasts to boost titres. CRV and CAV were harvested as cell-free virus from at least two 175 cm^2^ flasks when complete CPE was achieved. In the case of RL13^+^UL128L^+^ and RL13^−^UL128L^+^ viruses, low-titre CRV was concentrated 100-fold by centrifugation at 26 000 ***g*** for 1 h at 18 °C.

### Infectivity of HCMV stocks.

Titrations of cell-free virus were performed simultaneously in fibroblasts either in 30 mm plates or on glass coverslips. The cultures on 30 mm plates were fixed and stained for plaque counting at 12 days p.i. Those on coverslips were fixed at 3 days p.i. and permeabilized for IF analysis. The coverslips were treated with a mixture of antibodies recognizing the immediate-early UL123/UL122 proteins (IE1/IE2) (kindly provided by Edward Mocarski, Jr, Emory University School of Medicine, Atlanta, GA, USA) labelled using fluorescein isothiocyanate (FITC), and FITC-pre-conjugated antibody CCH2 (DAKO) recognizing the early UL44 protein (DNA polymerase processivity factor).

The ability of strain VR1814 at various passages in fibroblasts or endothelial cells to transfer by co-culture to leukocytes (leukocyte transfer) or endothelial cells (endotheliotropism) was monitored by using published methods (Gerna *et al.*, 2000, 2002a; Hahn *et al.*, 2004). Leukocyte transfer was determined by IF detection in leukocytes of the UL82 protein (pp65; expressed as the number of positive cells per 2×10^5^ leukocytes) via a cytospin preparation, and endotheliotropism was assessed from cell-free virus-induced CPE, with infection of 80–100 % of cells being scored as positive.

### Kinetics of virus growth.

The passages used were: AF1Rp38 (RL13^+^UL128L^+^), AF1Ep50 (RL13^−^UL128L^+^) and AF1Fp60 (RL13^−^UL128L^−^); U8Rp39 (RL13^+^UL128L^+^), U8Ep51 (RL13^−^UL128L^+^) and U8Fp54 (RL13^−^UL128L^−^); and U11Rp27 (RL13^+^UL128L^+^), U11Rp37 (RL13^−^UL128L^+^) and U11Fp51 (RL13^−^UL128L^−^). For each virus stock, one 35 mm plate containing 2×10^5^ fibroblasts was infected with 1 infectious unit of virus per cell (determined from IF titres). After adsorption for 1 h at 37 °C, unbound virus was removed by washing with medium and the cells were overlaid with 2 ml medium. Aliquots (1 ml) of medium were removed for titration of CRV in fibroblasts. Cells in these aliquots that had detached from the monolayer were pelleted by centrifugation at 500 ***g*** for 10 min at 18 °C and returned to the infected culture in 1 ml fresh medium. Virus yields were stored at −70 °C. At 8 days p.i., CAV was harvested by scraping the cells into 2 ml fresh medium and subjecting them to ultrasonic disruption.

## Figures and Tables

**Table 1.** Mutations that occurred during passage of strain AF1, with the final passages being F (p60), R (p50) and E (p50)

Location*	**Region†**	**Sequence‡**				**Coding effect**	**Dynamics§**	**Intermediate passages||**
		**N**	**F**	**R**	**E**			
10984	RL13	G	G	G	**A∧**	Termination	14/18	5 10 12 14 16
11460	RL13	C	**G∧**	C	C	Substitution	15/23	5 10 15 16 18 20
11488–11489	RL13	2	2	**Δ−**	2	Frameshift	30	10 20
11667	RL13	C	**T∧**	C	C	Termination	15/23	5 10 15 16 18 20
57436	UL44	[T]	**C=**	T	T	Substitution	50	5 12 25
105981	UL72	C	C	C	**T∧**	Substitution	30/30	5 12
106148	UL72	G	**A+**	**A+**	G	Substitution	60 (F); 50 (R)	None (F); none (R)
134792–134836	UL89 in	[45]	45	**Δ∧**	45	None	30/40	10 20 30
172215	UL123	[C]	C	C	**T=**	Substitution	50	10 20 30 40
172317	UL123	[C]	C	C	**T=**	Substitution	50	None
172375	UL123	[C]	C	C	**T=**	None	50	None
173346	UL123	[C]	C	C	**T=**	Substitution	46	10 20 30 40 42
173351	UL123	[C]	C	C	**T=**	Substitution	46	10 20 30 40 42
173582	UL123	[C]	C	C	**T=**	Substitution	44	10 20 30 40 42
173747	UL123 in	[C]	C	C	**T=**	None	46	10 20 30 40
177046	UL130	C	**A∧**	C	C	Termination	20/23	10 12 14 15 16 18 20
194890/944	*b*′	C	**A+**	C	C	None	60	None
194979/855	*b*′	C	**A=**	C	C	None	60	None
195025/809	*b*′	G	**C+**	G	G	None	60	None
195037/797	*b*′	C	C	C	**A=**	None	50	None
195049/785	*b*′	T	T	**G−**	**G=**	None	60 (R); 50 (E)	None (R); none (E)
195053/781	*b*′	T	**G−**	T	T	None	60	None
195056/778	*b*′	G	**C−**	G	G	None	60	None
195058/776	*b*′	T	**G−**	T	T	None	60	None
195081/753	*b*′	C	**A−**	C	C	None	60	None
195143/691/235892	*a*′	G	**T+**	G	G	None	60	None
196025/235010	*c*′	A	**C+**	A	A	None	60	None

*Location (nt) in the genome sequence. Multiple locations relate to duplications (*b*/*b*′ and *c*/*c*′) or triplications (*a*/*a*′/*a*) in the inverted repeats. †Regions affected by mutations are protein-coding or non-coding. The latter are located in the inverted repeats (*a*′/*b*′/*c*′) or introns (in) or are intergenic (IG). ‡N, Not passaged (clinical sample); F, passaged in fibroblast cells; R, passaged in epithelial cells; E, passaged in endothelial cells. The mutated nucleotide at each location is shown in bold, followed by a sign indicating relative abundance in the final passage: ∧, only the mutated nucleotide was detected; +, the mutated nucleotide predominated over the wild-type nucleotide; =, the mutated and wild-type nucleotides were present in approximately equal amounts; −, the mutated nucleotide was less abundant than the wild-type nucleotide. Where a deletion (Δ) occurred, the size (nt) is specified for the wild-type sequence. Where an insertion occurred, the original nucleotide is specified for the wild-type sequence and is followed by additional nucleotides (or replaced by the total number of nucleotides for larger insertions) for mutated sequences. Square brackets indicate that the sequence was not determined directly, but indirectly by reference to sequence alignments for other HCMV strains. nd, Passaging not done. §A single passage no. is that at which the mutation was first detected; a second passage no. is that at which only the mutation was detected. Square brackets (Table 4 only) indicate that the mutation detected in the E series was first detected during isolation in F cells. ||Passages analysed prior to that at which the mutation was first detected or that at which only the mutation was detected are listed. Later passages were also analysed.

**Table 2.** Mutations that occurred during passage of strain U8, with the final passages being F (p65), R (p51) and E (p51)

Location*	**Region†**	**Sequence‡**				**Coding effect**	**Dynamics§**	**Intermediate passages||**
		**N**	**F**	**R**	**E**			
11269–11270	RL13	2	2	**Δ−**	2	Frameshift	40	30 36 38
11890	RL13	A	A	A	**AA∧**	Frameshift	16/51	10 14 16 20 21 22 30
11924	RL13	G	**A∧**	G	G	Termination	16/20	11 12 14 16 18
35813	UL29	[A]	**G=**	A	A	Substitution	65	10 20 30 39 50
49479	UL36	[C]	C	C	**T+**	Substitution	10	10
49959	UL36	[C]	**CC=**	C	C	Frameshift	50	10 20 30 39
58564	UL45	[C]	C	**A−**	C	Substitution	40	20 30
100119	UL69	[C]	**T∧**	C	C	Substitution	20/65	10 20 30 39 50
106348	UL72	[G]	G	G	**A+**	Substitution	30	10 20
146722	UL100	[G]	**T=**	G	G	Substitution	65	10 20 30 39 50
146904	UL100	[C]	C	C	**T∧**	Substitution	39/50	10 20 30 39
149267	UL102	[T]	T	T	**C∧**	Substitution	30/50	10 20 30 40
178000	UL131A	C	**A∧**	C	C	Termination	18/20	10 12 14 15 16 18
181584	UL145	C	**G=**	C	C	Substitution	32	30
181906–182456	UL144	551	551	551	**Δ∧**	Deletion	30/51	30
182822	UL144/UL142 IG	C	**T=**	C	C	None	32	30
183281	UL144/UL142 IG	C	**T+**	C	C	None	32	30
183294	UL142	C	**T+**	C	C	Substitution	32	30
184383	UL141	C	**T+**	C	C	Substitution	32	9 20 30
186935	UL139/UL138 IG	[C]	**G=**	C	C	None	39	10 20 30
194454/1113	*b*′	T	**G∧**	T	T	None	65/65	None
194666/901	*b*′	C	**A∧**	C	C	None	65/65	None
194676/891	*b*′	T	**G=**	T	T	None	65	None
194677/890	*b*′	C	**A=**	C	C	None	65	None
194690/877	*b*′	C	**A+**	C	C	None	65	None
194749/818	*b*′	C	**A∧**	**A=**	C	None	65/65 (F); 51 (R)	None (F); none (R)
194774/793	*b*′	C	**A∧**	C	C	None	65/65	None
194792/775	*b*′	C	**A+**	C	C	None	65	None
194874/693/235647	*a*′	[G]	**C∧**	G	G	None	65/65	None

See Table 1 for footnotes.

**Table 3.** Mutations that occurred during passage of strain U11, with the final passages in series I being F (p63) and R (p52)

Location*	**Region†**	**Sequence‡**			**Coding effect**	**Dynamics§**	**Intermediate passages||**
		**N**	**F**	**R**			
**Series I**							
11378	RL13	A	**AA∧**	**AA∧**	Frameshift	8/9 (F); 30/52 (R)	4 6 7 8 (F); 25 28 30 33 35 37 39 40 (R)
83326	UL55	C	**T∧**	C	Substitution	58/62	20 30 40 45 50 58 60 61
176252	UL128	C	**A=**	**A–**	Termination	15 (F); 20 (R)	5 8 10 (F); 10 (R)
176307–176311	UL128	CAAGA	**TCTTG=**	**TCTTG−**	Substitutions	15 (F); 37 (R)	5 8 10 (F); 10 20 25 35 (R)
184505	UL141	G	**GT=**	**GT−**	Frameshift	63 (F); 52 (R)	None (F); none (R)
193985/735	*b*′	C	**A−**	C	None	63	None
194097/623	*b*′	T	**G=**	T	None	63	None
194099/621	*b*′	C	**A−**	**A=**	None	63 (F); 52 (R)	None (F); none (R)
194104/616	*b*′	C	**A−**	C	None	63	None
194144/576	*b*′	T	**G+**	**G+**	None	63 (F); 52 (R)	None (F); none (R)
194192/528	*b*′	T	**G−**	T	None	63	None
194216/504/234725	*a*′	T	**C=**	**C−**	None	63 (F); 52 (R)	None (F); none (R)
194220/500/234721	*a*′	G	**T+**	G	None	63	None
194222/498/234719	*a*′	A	**G=**	**G+**	None	63 (F); 52 (R)	None (F); none (R)
194263/457/234678	*a*′	T	**A−**	**A−**	None	63 (F); 52 (R)	None (F); none (R)
194268/452/234673	*a*′	G	G	**A−**	None	52	None
194795/234146	*c*′	G	**T=**	G	None	63	None
194829/234112	*c*′	C	**G+**	**G+**	None	63 (F); 52 (R)	None (F); none (R)
222612	US26	C	C	**T+**	Substitution	20	None
**Series II, which started with virus isolated in series I and shows mutations that occurred in RL13 and UL128L, with the final passage being F (p51)**							
10795	RL13	G	**T−**	nd	Termination	20	None
10976–10977	RL13	2	**Δ−**	nd	Frameshift	20	None
11378	RL13	A	**AA−**	nd	Frameshift	40	13
175795	UL128	C	**A∧**	nd	Not spliced	13/20	5 13 15
**Series III, which started with virus reisolation and shows mutations that occurred in RL13 and UL128L, with the final passage being F (p40)**							
11232–11233	RL13	2	**Δ−**	nd	Frameshift	21	10
11484	RL13	1	**Δ+**	nd	Frameshift	21	7 10
175739	UL128	G	**A∧**	nd	Termination	30/40	7 10 15 21 25 30 33

See Table 1 for footnotes.

**Table 4.** Mutations that occurred during passage of strain VR1814, with the final passages being F (p214) and E (p199)

Location*	**Region†**	**Sequence‡**			**Coding effect**	**Dynamics§**	**Intermediate passages||**
		**N**	**F**	**E**			
10999–12108	RL13 UL1	[1110]	**Δ+**	**Δ+**	Deletion	48 (F); [48] (E)	22 25 30 41 (F); none (E)
11775	RL13	[A]	**AA+**	**AA+**	Frameshift	48 (F); [48] (E)	22 25 30 41 (F); none (E)
28163–30607	UL23 UL24 UL25	[2445]	2445	**Δ+**	Deletion	199	None
41424	UL32	[G]	**A∧**	G	Substitution	214	None
51484	UL38	[G]	G	**GG=**	Frameshift	199	None
57447	UL44	[T]	T	**C=**	Substitution	199	None
73746	UL50	[C]	C	**CGAG+**	In-frame insertion	199	None
82573	UL55	[C]	**A=**	C	Substitution	214	None
82890	UL55	[T]	T	**C∧**	Substitution	199	None
85615	UL56	[C]	C	**T+**	Substitution	199	None
89021	UL57	[T]	T	**C∧**	Substitution	199	None
94456–94470	OriLyt	[15]	15	**Δ∧**	None	199	None
94474	OriLyt	[C]	**111∧**	**111∧**	None	214/214 (F); 199/214 (E)	None (F); none (E)
139372	UL89	[C]	**G+**	C	Substitution	214	None
145063	UL98	[A]	**C∧**	**C∧**	Substitution	214/214 (F); 199/214 (E)	None (F); none (E)
163725	UL112	[T]	T	**TGGT+**	In-frame insertion	199	None
171135	UL122	[G]	G	**A∧**	Substitution	199	None
176856	UL130	[G]	G	**T∧**	Substitution	199	None
177250	UL130	[A]	**G∧**	A	Substitution	48/48	22
177905	UL131A	[T]	**TT∧**	T	Frameshift	95/121	22 25 30 41 48 55 95
181943	UL145/UL144 IG	[T]	T	**TT+**	None	199	None
182580–185752	UL142 UL141 UL140	[3173]	**Δ∧**	**Δ∧**	Deletion	48/143 (F); [48]/199 (E)	22 25 30 41 48 55 95 121 (F); none (E)
194218/1118	*b*′	[C]	C	**A∧**	None	199	None
194225/1111	*b*′	[T]	**G−**	T	None	214	None
194290/1046	*b*′	[C]	C	**A+**	None	199	None
194352/984	*b*′	[C]	**A−**	C	None	214	None
194367/969	*b*′	[C]	C	**A∧**	None	199	None
194389/947	*b*′	[C]	**A−**	**A+**	None	214 (F); 199 (E)	None (F); none (E)
194498/838	*b*′	[C]	C	**A∧**	None	199	None
194563/773	*b*′	[C]	**G−**	C	None	214	None
195182/154/234767	*a*′	[T]	**G−**	**G∧**	None	214 (F); 199 (E)	None (F); none (E)
195286/50/234663	*a*′	[A]	A	**C∧**	None	199	None
195477/234472	*c*′	[A]	**C=**	**C=**	None	214 (F); 199 (E)	None (F); none (E)
195488/234461	*c*′	[C]	**A−**	C	None	214	None
195545/234404	*c*′	[G]	**T+**	**T∧**	None	214 (F); 199 (E)	None (F); none (E)
195552/234397	*c*′	[A]	**C−**	**C∧**	None	214 (F); 199 (E)	None (F); none (E)
195703/234246	*c*′ (IRS1)	[A]	**C∧**	**C+**	Substitution	214 (F); 199 (E)	None (F); none (E)
195709–197642	IRS1	[1934]	1934	**Δ∧**	Deletion	199	None
198925	US1/US2 IG	[T]	T	**C=**	None	199	None
225998	US28	[T]	T	**TTT+**	Frameshift	199	None
230525	US34	[G]	G	**GAG=**	Frameshift	199	None

See Table 1 for footnotes.
